# Retrospective analysis of the efficacy of low-intensity extracorporeal shock wave therapy on young and middle-aged patients with erectile dysfunction responsive to PDE5Is: reducing the use of PDE5Is

**DOI:** 10.1093/sexmed/qfae065

**Published:** 2024-09-29

**Authors:** Rui-Jie Yao, Mao-Yuan Wang, Qiang Chen, Hong Xiao, Peng Yang, Yi-Lang Ding, Xi Chen, Song-Xi Tang, Hui-Liang Zhou

**Affiliations:** Department of Andrology and Sexual Medicine, The First Affiliated Hospital of Fujian Medical University, Fuzhou 350005, China; Department of Andrology and Sexual Medicine, The First Affiliated Hospital of Fujian Medical University, Fuzhou 350005, China; Department of Andrology and Sexual Medicine, The First Affiliated Hospital of Fujian Medical University, Fuzhou 350005, China; Department of Andrology and Sexual Medicine, The First Affiliated Hospital of Fujian Medical University, Fuzhou 350005, China; Department of Andrology and Sexual Medicine, The First Affiliated Hospital of Fujian Medical University, Fuzhou 350005, China; Department of Andrology and Sexual Medicine, The First Affiliated Hospital of Fujian Medical University, Fuzhou 350005, China; Department of Andrology and Sexual Medicine, The First Affiliated Hospital of Fujian Medical University, Fuzhou 350005, China; Department of Andrology and Sexual Medicine, The First Affiliated Hospital of Fujian Medical University, Fuzhou 350005, China; Department of Andrology and Sexual Medicine, The First Affiliated Hospital of Fujian Medical University, Fuzhou 350005, China

**Keywords:** low-intensity extracorporeal shock wave therapy, erectile dysfunction, phosphodiesterase type 5 inhibitors

## Abstract

**Background:**

Low-intensity extracorporeal shock wave therapy (Li-ESWT) is a new method for treating erectile dysfunction (ED), but there are no standards yet for its indications.

**Aim:**

The study aimed to suggest the early clinical efficacy of Li-ESWT and explore its related factors in young and middle-aged patients with ED who responded to phosphodiesterase type 5 Inhibitors (PDE5Is).

**Methods:**

Data from 61 patients with ED who had previously responded to oral PDE5Is and subsequently underwent Li-ESWT were collected. This included information on age, body mass index, total testicular volume, sex hormones, as well as IIEF-EF scores before treatment and at 1, 3, and 6 months after treatment. The treatment regimen involves a weekly session for four consecutive weeks, with each session administering 5000 shock wave pulses. Linear regression analysis was utilized to identify factors associated with the efficacy of Li-ESWT treatment. Additionally, the improvement in different severity groups of ED before and after treatment, along with their IIEF-EF scores, was compared.

**Outcomes:**

Li-ESWT was more targeted and effective for young and middle-aged patients with erectile dysfunction who responded to PDE5Is.

**Results:**

The age of enrolled patients ranges from 22 to 53 years old, and the IIEF-EF scores at 1 month, 3 months, and 6 months after treatment were compared to baseline for efficacy assessment, showing significant improvements (*P* < .0001) in all instances. Linear regression analysis using baseline data revealed predictive factors associated with treatment efficacy: treatment efficacy was negatively correlated with baseline IIEF-EF scores (*t* = −2.599, *P* = .013) and positively correlated with baseline LH levels (*t* = 2.170, *P* = .036).

**Clinical Implications:**

Given the considerable cost of Li-ESWT treatment and the emphasis on treatment continuity, we hope to identify the most suitable candidates for Li-ESWT therapy, thereby optimizing its application.

**Strengths and Limitations:**

Our findings provide a better solution for nonelderly ED patients who are responsive to PDE5Is. This study was limited by our sample size and follow-up time.

**Conclusion:**

After 3 months of Li-ESWT, the IIEF-EF score gradually stabilizes and short-term maintenance of PDE5Is medication increases the responsiveness to shock wave therapy.

## Introduction

Erectile dysfunction (ED) refers to the inability of males to achieve and maintain a sufficient penile erection for satisfactory sexual intercourse.^1^ The European Male Aging Study indicates that the prevalence of ED is approximately 30% to 65%, with higher rates among elderly males.^2^ Although phosphodiesterase type 5 inhibitors (PDE5Is) are the most common first-line treatment for ED, some patients are unable to adhere to medication or discontinue it for reasons such as lack of sexual opportunities or desire, lower-than-expected efficacy, high costs, and insufficient emotional preparation for resuming sexual activity after a prolonged period of abstinence, either by the partner or by the patient.^3-5^

Low-intensity extracorporeal shock wave therapy (Li-ESWT) is a novel approach in recent years for treating ED, with most studies confirming its efficacy in treating ED, particularly in patients with mild to moderate ED and vascular ED.^6^^,^^7^ Its primary mechanism of action is to stimulate the expression of vascular endothelial growth factor (VEGF), thereby promoting the regeneration of penile blood vessels to improve ED.^7^^,^^8^ Multiple systematic reviews and randomized controlled trials have focused on the general applicability of Li-ESWT in the treatment of ED, all demonstrating good efficacy.^6^^,^^9-11^ Additionally, some studies suggest that Li-ESWT has a synergistic effect with PDE5Is, meaning that simultaneous use of PDE5Is after Li-ESWT can enhance treatment effectiveness.^12^^,^^13^ However, it is currently unknown whether ED patients responsive to PDE5Is can reduce or discontinue PDE5Is usage through Li-ESWT. This study aims to evaluate the efficacy of Li-ESWT in middle-aged and young ED patients responsive to PDE5Is using the International Index of Erectile Function-Erectile Function (IIEF-EF) score and attempts to address this question.

## Participants and methods

### Study population

This study included 61 cases of young and middle-aged patients with ED who had multiple visits to the Department of Andrology, from January 2020 to May 2023. Patients underwent routine physical examinations, and information including age, body mass index (BMI), total testicular volume, hormone levels, comorbidities, and usage of PDE5Is was collected.

Inclusion criteria for Li-ESWT were as follows: ED patients with a disease course of more than 3 months, aged from 20 to 55, who respond to PDE5Is and have stable sexual partners, should engage in regular sexual activity at least once a week before treatment, during treatment, and throughout the follow-up period. However, in actual sexual encounters, patients who respond to PDE5is may express a desire to reduce or discontinue their use if the effects do not meet their psychological expectations, or if they are concerned about potential long-term adverse effects and drug dependence. Before LI-ESWT treatment, they discontinued the medication for at least 3 months.

Exclusion criteria: (1) patients with congenital malformations or abnormalities of the genital organs, (2) patients with ED caused by spinal cord injury, (3) patients with penile fibrosis (Peyronie’s disease), (4) patients with a history of penile or pelvic organ trauma or surgery, and (5) patients with a history of coagulation disorders, anticoagulant or antiplatelet therapy, or other bleeding disorders.

The study included 61 young and middle-aged patients aged between 22 and 53 years, with a mean(± standard deviation) age of 35.44(±6.57) years. All patients had been orally taking PDE5Is such as Sildenafil and Tadalafil before treatment. Erectile function was assessed using the IIEF-EF score.^14^ Patients were initially screened using the IIEF-5 questionnaire, and based on their IIEF-EF scores after discontinuing PDE5Is, the presence and severity of ED were determined as follows: scores of 26–30 indicated no ED, 17–25 indicated mild ED, 11–16 indicated moderate ED, and ≤10 indicated severe ED.^15^ (Subsequently, the IIEF-EF scores before treatment, and at 1, 3, and 6 months after treatment were abbreviated as EF0, EF1, EF3, and EF6, respectively.) Prior to Li-ESWT treatment, the distribution of IIEF-EF scores was as follows: 30 cases with mild ED (scores of 17–25), 12 cases with moderate ED (scores of 11–16), and 19 cases with severe ED (scores of 1–10) (Table 1). Among them, one case had diabetes, with well-controlled blood sugar levels and no secondary complications.

**Table 1:** Clinical data of enrolled patients.

Age, year (mean ± SD)	35.48 ± 6.61
BMI, Kg/m^2^ (mean ± SD)	24.29 ± 3.07
Total testicular volume, mL (mean ± SD)	28.87 ± 6.70
FSH, IU/L median (interquartile range)	4.83 (3.09, 6.09)
LH, IU/L (mean ± SD)	4.16 ± 1.94
E2, pmol/L (interquartile range)	105 (76.53, 140.30)
T, nmol/L (mean ± SD)	18.14 ± 6.64
PRL, mIU/L (mean ± SD)	249.48 ± 106.36
EF0, scores (mean ± SD)	15.07 ± 7.38
IIEF-EF scores	
Scores of 17–25, *n* (percentage)	30 (49.18%)
Scores of 11–16, *n* (percentage)	12 (19.67%)
Scores ≤10, *n* (percentage)	19 (31.15%)

Abbreviations: BMI, body mass index; FSH, follicle-stimulating hormone; LH, luteinizing hormone; E2, estradiol; PRL, prolactin; EF0, IIEF-EF scores of patients before Li-ESWT treatment.

### Li-ESWT

The linear low-energy shock wave therapy device (origin: Israel; model: RENOVA；manufacturer: Initia Lid; manufacturer location: 68 Amal SL(P.O. Box 4159).49513 Petah Tikva, Israel) is a mobile electromagnetic linear shock wave therapy device, consisting of a mechanical and an electrical module including a transducer (electromagnetic head). The treatment regimen involves a weekly session for four consecutive weeks, with each session administering 5000 shock wave pulses. This includes 1600 pulses each to the left and right crus penis, and 900 pulses each to the left and right corpus cavernosum. The energy density is 0.09 mJ/mm^2^, and the frequency is 150 pulses per minute. A total of 20 000 shock wave pulses are administered in a single 4-week course of treatment.

### Following-up

After completing a course of treatment, patients were followed up at 1, 3, and 6 months. During and after the treatment, patients were instructed to maintain regular sexual activity of at least once a week. Additionally, patients were required to use a low dose of PDE5Is daily (Sildenafil 25 mg once daily or Tadalafil 5 mg once daily), which they purchased on their own. Those in the mild ED group (*n* = 30) continuing medication until 1 month after treatment; others in the moderate to severe ED groups (*n* = 31) continuing until 3 months after treatment, and whether to continue medication depends on their individual sexual activity. Erectile function was evaluated using the IIEF-EF score at different follow-up times. All 61 patients were followed up at least once (1 month after treatment), 59 patients were followed up twice (3 months after treatment), and 34 patients were followed up three times (6 months after treatment).

### Statistical methods

Data were statistically analyzed using IBM SPSS Statistics (Version 26). Normally distributed data were expressed as mean ± standard deviation ($\bar{x}$ ± S), while non-normally distributed variables were expressed as median (25% and 75% interquartile range). Categorical variables were presented with number (percentage). The Shapiro–Wilk test was used to assess the normality of data distribution, and Levene’s test was used to evaluate homogeneity of variance. For normally distributed data with homogeneity of variance, comparisons between groups were performed using the *t*-test. Non-normally distributed continuous variables and categorical data were compared using the Wilcoxon signed-rank test. Multiple linear regression analysis was employed to examine the correlation between factors such as age, BMI, testicular volume, hormone levels, and pretreatment IIEF-EF score with treatment efficacy. All statistical tests were two-tailed, and *P*-values<0.05 were considered statistically significant.

## Results

**Figure 1:**
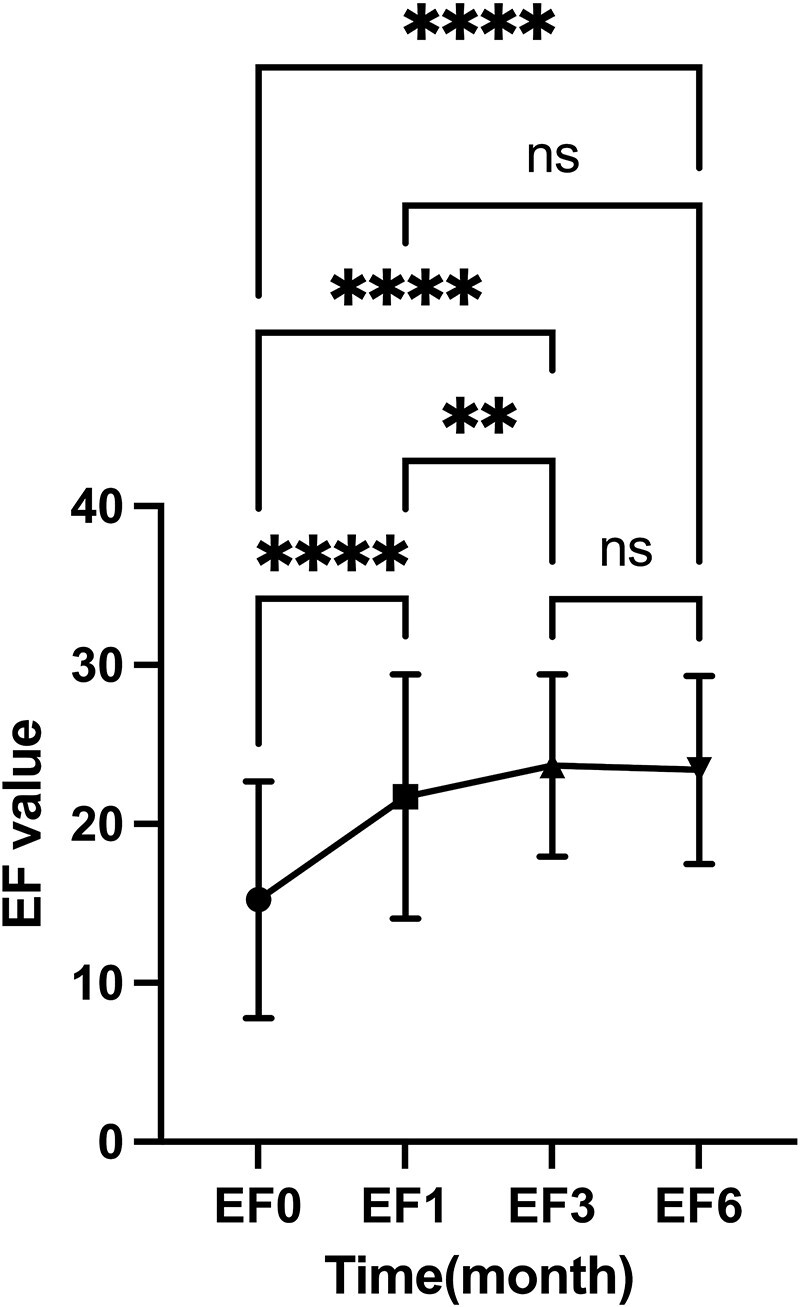
Overall changes in IIEF-EF scores before and after Li-ESWT treatment: one-way ANOVA and mixed effects model. Abbreviation: ns, not significant. ^*^^*^P < .01, ^*^^*^^*^^*^P < .0001.

**Figure 2:**
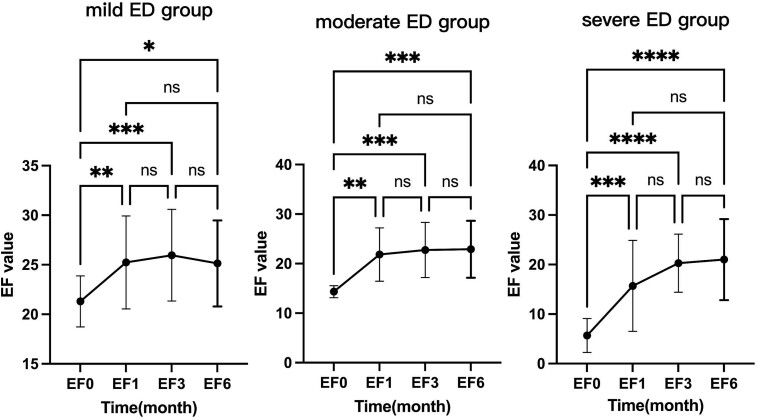
Comparison of IIEF-EF scores at 1 month, 3 months, and 6 months after treatment in different severity levels of ED: one-way ANOVA and mixed effects model. Abbreviation: ns, not significant. ^*^P < .05, ^*^^*^P < .01, ^*^^*^^*^P < .001, ^*^^*^^*^^*^P < .0001.

The baseline IIEF-EF score of the patients in this group was 15.26 ± 7.47. The IIEF-EF scores at 1, 3, and 6 months after treatment were 21.73 ± 7.69, 23.68 ± 5.73, and 23.40 ± 5.93, respectively, all showing significant improvement compared to baseline (*P* < .0001, Table 2). Specifically, there was a rapid increase in the IIEF-EF score at 1 month after treatment (*P* < .01), followed by a further increase at 3 months after treatment compared to 1 month (*P* < .01). Although there was a slight decrease in the IIEF-EF score at 6 months after treatment compared to 3 months, it was not statistically significant (*P* > .05). This indicates that the improvement in erectile function persisted at 1 and 3 months after Li-ESWT treatment and could be maintained up to 6 months after treatment (Figure 1). Further analysis revealed that this trend was also observed in patients with mild, moderate, and severe ED (Figure 2). The improvement in IIEF-EF scores at 1 and 3 months after treatment was more significant in the severe ED group compared to the mild and moderate groups, although it did not reach normal levels.

**Table 2:** Comparison of IIEF-EF scores in patients before and after Li-ESWT treatment.

	EF0	EF1	EF3	EF6
IIEF-EF scores	15.07 ± 7.38	21.59 ± 7.68^a^	23.58 ± 5.72^a^	23.38 ± 6.02^a^

a Wilcoxon signed-rank test for nonparametric analysis *P*<.0001

Abbreviations: the IIEF-EF scores before treatment, and at 1, 3, and 6 months after treatment were abbreviated as EF0, EF1, EF3, and EF6.

**Figure 3:**
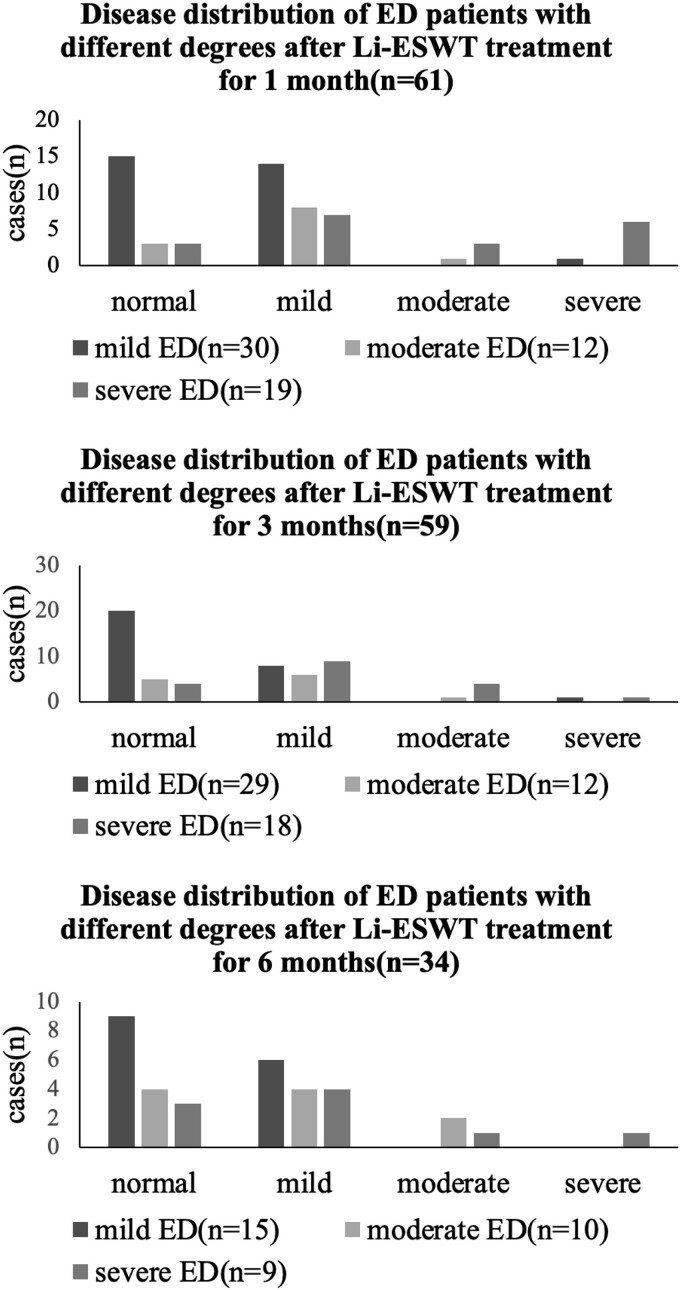
Distribution of patients with different severity levels of ED after Li-ESWT treatment at 1 month, 3 months, and 6 months: The proportion of patients recovering to normal in the mild ED group was the highest at 1 month (15/30, 50.00%).

In this group, there were 30 cases classified as mild ED (scores of 17–25), 12 cases as moderate ED (scores of 11–16), and 19 cases as severe ED (scores of 1–10) according to the Li-ESWT pretreatment IIEF-EF scores. After receiving one course of Li-ESWT treatment, the proportion of cases with IIEF-EF scores ≥26 significantly increased at 1, 3, and 6 months, accounting for 34.43% (21/61), 49.15% (29/59), and 47.06% (16/34), respectively. This increase was mainly observed in patients with mild ED, while the number of cases with moderate and severe ED transitioning to mild ED also increased significantly, with some patients achieving an IIEF-EF score of 26 or above (Figure 3).

**Figure 4:**
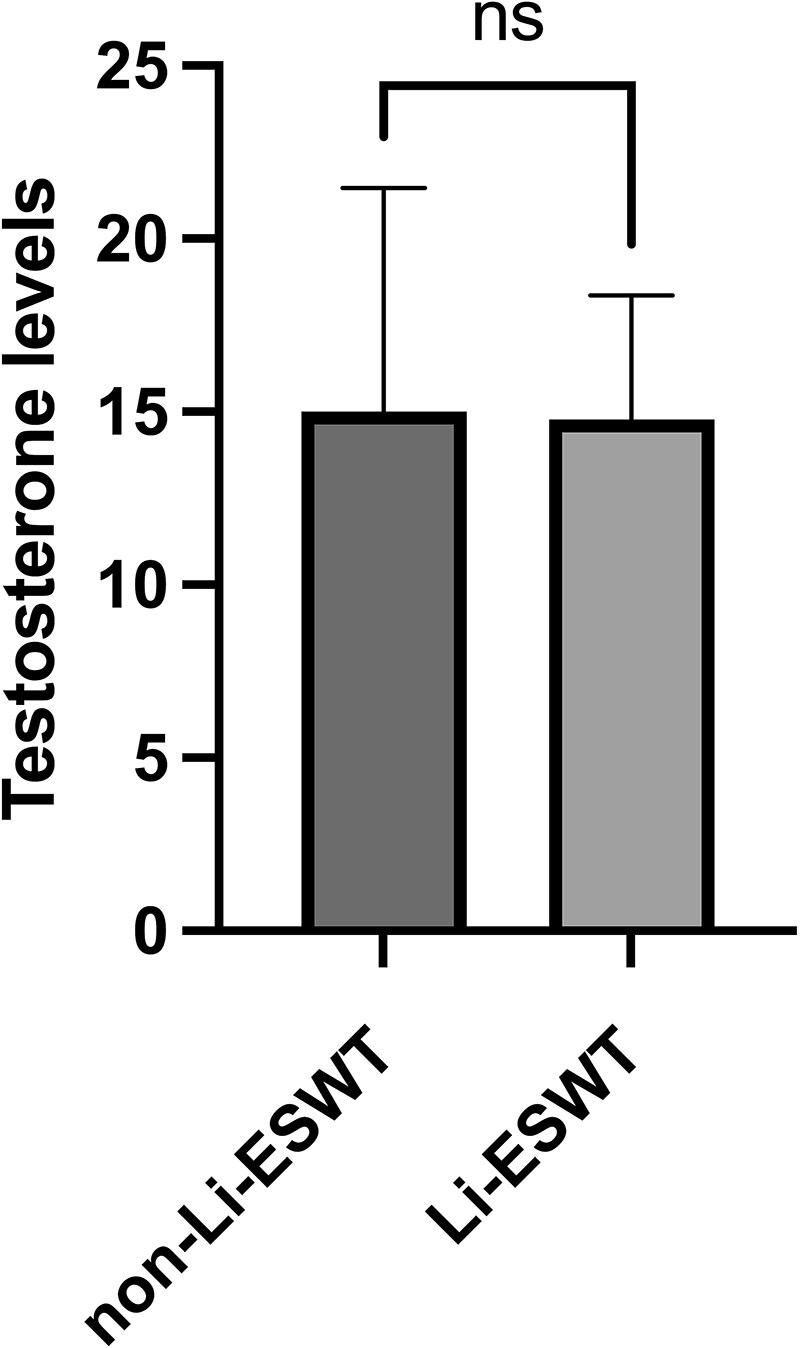
Change of testosterone levels in some patients before and after Li-ESWT treatment: Welch’s *t*-test. Abbreviation: ns, not significant.

Multiple linear regression analysis was conducted to assess the correlation between patient age, BMI, total testicular volume, hormone levels, and pretreatment IIEF-EF score with the overall efficacy of Li-ESWT treatment. The results indicated that LH levels and the EF0 parameter were significantly correlated with the overall efficacy of Li-ESWT treatment. Specifically, LH levels were positively correlated with treatment efficacy, indicating that higher LH levels were associated with more significant improvements in IIEF-EF scores after treatment. Conversely, the pretreatment IIEF-EF score was negatively correlated with treatment efficacy, with lower pretreatment IIEF-EF scores associated with more significant improvements in IIEF-EF scores after treatment (Table 3). Additionally, testosterone levels were measured in 21 patients before and after Li-ESWT treatment, and paired data comparison showed no statistically significant difference (*P* = 0.85, Figure 4).

**Table 3:** Linear regression analysis of baseline data.

Independent variables	*B*	*Beta*	*F*	*R^2^* (Adjusted *R^2^*)	*t*	*P*
Constants	29.044		2.680^a^	0.359 (0.225)	2.301	0.026
Age	−0.189	−0.184			−1.169	0.249
BMI	−0.600	−0.252			−1.699	0.097
Total testicular volume	0.063	0.056			0.415	0.680
FSH	−0.197	−0.092			−0.092	0.569
LH	1.191	0.324			2.170	0.036
E2	0.009	0.079			0.556	0.581
T	−0.069	−0.152			−0.450	0.655
PRL	−0.006	−0.085			−0.562	0.577
EF0	−0.347	−0.368			−2.599	0.013

a Multiple linear regression analysis *P* < .01

Abbreviations: BMI, body mass index; FSH, follicle-stimulating hormone; LH, luteinizing hormone; E2, estradiol; PRL, prolactin; EF0, IIEF-EF scores of patients before Li-ESWT treatment.

Among the 61 patients, no adverse reactions or complications occurred during the course of Li-ESWT treatment and follow-up period. Only 14 moderate to severe ED patients continued with on-demand PDE5Is use in the sixth month. Most patients had good tolerance for PDE5Is. A few patients experienced transient side effects such as nasal congestion or facial redness while using Tadalafil or Sildenafil, but these were temporary, did not cause lasting discomfort, and affect the Li-ESWT treatment.

## Discussion

Previous studies have indicated that Li-ESWT can improve local blood supply, but the specific mechanism is still not conclusively determined. However, it is relatively clear that *ex vivo* studies and animal experiments have demonstrated that SWT can promote the formation of new blood vessels and stimulate the regeneration of damaged nerve fibers.^16^ Li-ESWT has a significant promoting effect on the expression of vascular endothelial growth factor (VEGF) in the corpus cavernosum,^17^^,^^18^ leading to improvement in erectile function. In recent years, several systematic reviews and randomized controlled trials^6^^,^^9-11^ have elucidated the precise efficacy of Li-ESWT in treating ED. This noninvasive treatment modality has provided significant benefits to patients, especially those with vascular/organic ED.

In our study, shock wave therapy for ED also yielded satisfactory results. The IIEF-EF scores showed a continuous improvement process at 1 and 3 months after Li-ESWT treatment, with most patients gradually stabilizing after 3 months. By 6 months, there was no significant change compared to the scores at 3 months, indicating that patients reached a stable phase of improvement at 3 months after Li-ESWT treatment. Additionally, during Li-ESWT treatment and in the 1-3 months post-treatment period, we continued to recommend daily use of low-dose PDE5Is (Sildenafil 25 mg or Tadalafil 5 mg q.n) to increase the responsiveness to shock wave therapy. Studies have reported that Li-ESWT increases the production of cGMP, which is the second messenger necessary for smooth muscle relaxation in penile erection; meanwhile, PDE5Is such as Sildenafil and Tadalafil inhibit cGMP degradation,^19^ synergistically accelerating penile vascular remodeling alongside Li-ESWT. Therefore, maintaining short-term PDE5Is treatment post-Li-ESWT can expedite patient benefit from the therapy and increase drug sensitivity of PDE5Is, ultimately allowing most mild ED patients to discontinue on-demand medication while enabling moderate and severe ED patients to reduce their dosage or transition towards withdrawal over time. The study specifically selected ED patients who responded well to PDE5Is due to their predominantly vascular/organic ED nature, making them more suitable for Li-ESWT—an innovative point we aim to emphasize.

Based on the proportion of individuals achieving normal IIEF-EF scores after Li-ESWT treatment, it is evident that the majority of patients with mild ED achieved normalization, indicating the highest probability of improvement for this subgroup. Additionally, some patients with moderate to severe ED also experienced improvements to normal levels. The pretreatment IIEF-EF score reflects the severity of the initial ED condition, with larger scores correlating with smaller improvements. Patients with slightly higher scores, such as those with mild ED, may experience limited room for improvement, whereas those with severe ED may experience the opposite. Liang et al.^9^ conducted a meta-analysis on the treatment of erectile dysfunction with Li-ESWT, showing that patients with moderate/severe ED experienced significant improvements in IIEF-EF scores after treatment, which aligns with the results of our study. The severity of ED correlated with the extent of improvement after treatment, with more significant improvements observed in patients with more severe ED. Although there was a slight overall decrease in IIEF-EF scores in the mild ED group at 6 months after treatment, this change was not statistically significant. We attribute this to a decrease in compliance or loss to follow-up among some patients who experienced improvement or cure.

The age range of patients in our study was 22–53 years old, and there was no significant difference in improvement among different age groups, indicating that Li-ESWT yielded satisfactory results in the treatment of middle-aged and young patients, suggesting its applicability to a wide range of individuals. Currently, regarding the association between sex hormones and ED, some cohort studies^20^^,^^21^ suggest that total testosterone, bioavailable testosterone, sex hormone-binding globulin, and ED are not correlated. The interaction between LH and total testosterone indicates that only when LH levels are 6 IU/L or higher, the probability of ED decreases more rapidly with increasing total testosterone levels. Our data also align with this conclusion. Among various sex hormone indicators such as PRL, FSH, LH, E2, and T, only LH values were found to be positively correlated with treatment efficacy. We speculate that within the normal reference range, an increase in LH values was associated with more significant improvements in IIEF-EF scores after treatment. Other sex hormones, such as PRL and E2, besides hyperprolactinemia leading to decreased libido and sexual dysfunction through hypothalamic–pituitary–gonadal axis regulation, have not been clearly implicated in directly causing ED. Factors such as testicular volume should be analyzed in conjunction with testosterone levels. Studies by Galdiero et al.^22^ suggested that testosterone decline associated with PRL may serve as a secondary factor contributing to ED. An imbalance in the ratio of E2 to T may reduce the pressure of the corpus cavernosum smooth muscle mediated by NO,^23-25^ playing a role in the pathogenesis of ED, but further research is needed.

As far as the safety of Li-ESWT is concerned, our study has not encountered any adverse reactions or complications in patients with ED after Li-ESWT treatment so far, and there were no significant differences in testosterone levels before and after treatment. Recently, prospective controlled studies^26^ have also suggested that Li-ESWT treatment has no significant impact on reproductive function and hormone levels in patients with ED and Peyronie’s disease. It is reasonable to believe that exposing the penis to low-intensity shock waves is safe for the testicles; hence, we selected a low-energy density of 0.09 mJ/mm^2^ for treatment.

In summary, the highlight of our study lies in selecting middle-aged and young ED patients who are responsive to PDE5Is for Li-ESWT, further confirming the unique advantages of Li-ESWT in treating vascular ED and exploring factors associated with treatment efficacy. The critical limitations of this study are the initially low proportion of patients who underwent hormone screening and a high rate of loss to follow-up at 6 months. Additionally, this was a retrospective study without an a priori protocol. Further discussion is needed regarding the role of Li-ESWT in improving confidence in the psychogenic ED. As Li-ESWT treatment is still in the exploratory stage and not yet widely used, limited by our sample size and follow-up time, future studies should include larger sample sizes to observe long-term efficacy changes. Further investigations into the efficacy of Li-ESWT in patients with comorbidities and in middle-aged to elderly patients will be necessary to better understand the treatment indications of Li-ESWT and standardize the treatment of ED patients.

## Conclusion

Low-intensity extracorporeal shock wave therapy is more targeted and effective for young and middle-aged patients with erectile dysfunction who respond to PDE5Is. After 3 months of treatment, the IIEF-EF score gradually stabilizes. Short-term maintenance of PDE5Is medication increases the responsiveness to shock wave therapy. Pretreatment IIEF-EF scores and LH levels are correlated factors for efficacy. Patients with mild ED have a greater advantage in returning to normal after treatment.
